# Synthesis, Spectroscopic, Thermal, and Catalytic Properties of Eight New Complexes of Metal(II) Formates or Propionates with Imidazole; Relationship between the Carbon Chain Length and Catalytic Activity

**DOI:** 10.3390/ma15010142

**Published:** 2021-12-25

**Authors:** Bartłomiej Rogalewicz, Tomasz Maniecki, Radosław Ciesielski, Agnieszka Czylkowska

**Affiliations:** Institute of General and Ecological Chemistry, Faculty of Chemistry, Lodz University of Technology, Zeromskiego 116, 90-924 Lodz, Poland; tomasz.maniecki@p.lodz.pl (T.M.); radoslaw.ciesielski@p.lodz.pl (R.C.)

**Keywords:** metal(II) complexes, imidazole, formates, propionates, TG-DTG, FTIR, catalyst, styrene oxidation

## Abstract

In one of our previously published articles, we reported the synthesis, spectroscopic, thermal, and catalytic properties of four new M(II) acetate (where M = Co, Ni, Cu, Zn) complexes with imidazole. Presented compounds exhibited activity in the reaction on catalytic oxidation of styrene. In this study we have synthesized and investigated properties of analogous compounds, however using formates or propionates of mentioned metal cations instead of acetates. Such an approach allowed us to draw valuable conclusions concerning the relationship between the carbon chain length and catalytic activity, which is an important factor for catalyst modeling. Synthesized compounds have been thoroughly investigated using appropriate analytic techniques: AAS (Atomic Absorption Spectrometry), FTIR (Fourier-Transform Infrared Spectroscopy), and TGA (Thermogravimetric Analysis). Catalytic properties have been studied under the same previous conditions, using GC-FID (GC-chromatograph equipped with FID detector).

## 1. Introduction

Incorporation of different ligands or counterions can significantly change physicochemical or biological properties of metal-based coordination compounds. One of the most popular ligands is imidazole. Imidazole and its derivatives are also known for their valuable properties, e.g., antifungal, antibacterial, anti-inflammatory, antiviral, and anticancer [1,2,3,4,5,6,7]. Imidazole itself is also a part of biologically important systems, e.g., one of the amino acids—histidine. Enormous variety of possible connections of different metal centres and compounds exhibiting useful properties or their derivatives create great opportunity to obtain outstanding results. It is thus important to determine the relationship between structure and activity, which is currently one of the most promising strategies in new drugs and catalyst design. All four mentioned metals, cobalt [8,9,10,11], nickel [12,13,14], copper [15,16,17], and zinc [17,18,19] are crucial and widely used in catalysis. One of the important reactions, problematic from an industrial point of view, is the reaction of the oxidation of styrene. This reaction is usually carried out in the presence of peracids however, since it leads to obtaining undesirable products, an ecological oxidizer (H_2_O_2_) can be used instead. In order to ensure proper conversion, a catalyst has to be used. Some catalysts have already been tested, like chromium-silica systems—resulting in obtaining benzaldehyde as the main product [20] or molecular sieves—in this case the main product was phenylacetaldehyde [21]. Benzaldehyde was also obtained in many other cases. Some examples comprise of modified cobalt or zinc oxide catalysts [22,23], as well as copper(II) complexes [24].

In one our previously published articles, we reported the synthesis, spectroscopic, thermal, and catalytic properties of four new M(II) acetate (where M = Co, Ni, Cu, Zn) complexes with imidazole [25]. Apart from experimental methods, such compounds can also be studied using computational techniques [26,27,28]. After physicochemical characterisation, synthesized compounds have additionally been tested for catalytic activity in a styrene oxidation reaction. Performed tests have given promising results with good percentage styrene conversion and almost 100% selectivity towards carbon dioxide formation. This is an important fact since one of the possible, undesired products for this reaction is benzaldehyde. In this paper, we report the synthesis and study of the physicochemical properties of analogous compounds, however using the formates or propionates of mentioned metal cations instead of acetates. Such an approach allows us to draw some valuable conclusions about the relationship between the carbon chain length and catalytic activity of investigated compounds, as well as thoroughly investigate their properties. (Abbreviations: L_1_—formate anion; Ac—acetate anion; L_2_—propionate anion; Im—imidazole; Im_d_—deprotonated imidazole)

## 2. Materials and Methods

All chemicals that were used for the synthesis were purchased from the following companies: Sigma-Aldrich, Pol-Aura, and Avantor Performance Materials Poland and were used without further purification. The contents of Co(II), Ni(II), Cu(II), and Zn(II) ions were determined by Atomic Absorption Spectrometry. Standard solutions from Merck (1000 mg/L, Darmstadt, Germany) were used for the preparation of diluted solutions used for calibration. For analysis, distilled water (electrical conductivity 0.05 µS) was used (obtained with Polwater system). Infrared spectra were recorded using IRTracer-100 Schimadzu Spectrometer (Japan) (3200–600 cm^−1^, accuracy of recording: 1 cm^−1^) in potassium bromide pellets. Thermal decompositions of compounds were studied using the TG-DTG thermogravimetric technique (range of temperature 25–800 °C; heating rate: 10 °C·min^−1^). TG and DTG curves were recorded using Netzsch TG 209 (Germany), in ceramic crucibles, under air atmosphere, v = 20 mL·min^−1^. As a reference material, ceramic crucibles were used. Catalytic properties have been studied in the liquid-phase styrene oxidation reaction. Reactions were carried out in 50-mL round-bottom flasks with the molar ratio of reagents being the same as in the previous part of the study [25]: C_2_H_3_N:H_2_O_2_:C_8_H_8_ = 1:1:1. The reaction mixture was stirred in a water bath at 60 °C and refluxed. After reaching this temperature, 10 mg of the catalyst was added. The optimum time of reaction for obtaining reliable results was found to be 2 h. After that time, the analysis of styrene content was performed using gas chromatography with a flame ionization detector (GC-FID, HP 5890, Hewlett Packard Corporation, (USA). The optimal parameters of method found for our study were as follows: Separation column: ZB-FFAP capillary column (30 m × 0.25 mm × 0.25 µm) with oven parameters: 60 °C for 8 min to 150 °C/min for 4 min; injection temperature of 225 °C; injection split: 11:8:1, 0.5 µL; and detector temperature of 250 °C. A carrier gas helium was used with a flow of 3.4 mL/min. As previously done, conversion degrees were determined according to the formula below:

$$\mathrm{Conversion}=\left[\frac{A_{0}-A_{t}}{A_{0}}\right]\times 100\%$$

where: A_0_ is the initial styrene concentration in reactant mixture and A_t_ is the concentration of styrene after 2 h of the reaction.

### 2.1. Synthesis of M(II) Formates and Propionates

**Figure 1:**
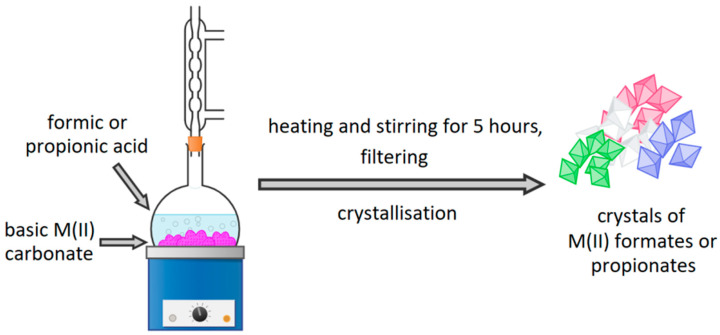
Synthesis of appropriate M(II) formates and propionates.

M(II) formates and propionates for synthesis were prepared using concentrated formic and propionic acids. Mixtures of appropriate acid and basic M(II) carbonate have been heated, refluxed, and stirred for 5 h. After that time, the excess of the unreacted carbonates was filtered. Filtrates were cooled and left for several days for crystallization. Obtained crystals were filtered and ground in a mortar. The purity of synthesized compounds was proven with thermogravimetric analysis. It also allowed establishing the amount of water molecules in hydrated compounds. Synthesis of M(II) formates and propionates is shown in Figure 1.

### 2.2. Synthesis of M(II) Formate and Propionate Complexes with Imidazole

**Figure 2:**
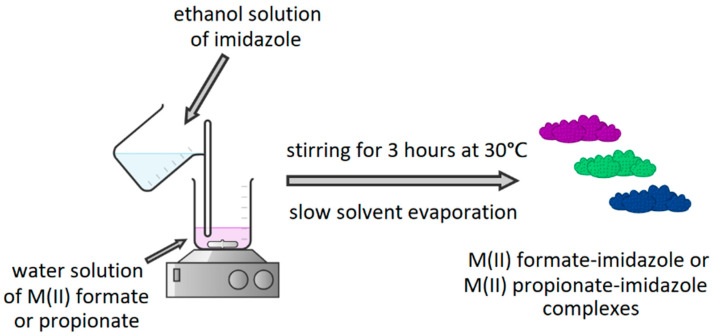
Synthesis of M(II) formate-imidazole or M(II) propionate-imidazole complexes.

In the next step, complexes of M(II) formates and propionates with imidazole were synthesized. Appropriate M(II) formate or propionate was dissolved in distilled water and mixed with an ethanol solution of imidazole. Reaction mixtures were heated to 30 °C and stirred for 3 h. The volume of the reaction mixture did not exceed 60 mL. Figure 2 presents a synthesis of the described complexes.

The molar ratio of metal cation and imidazole was determined based on the formulas of the complexes synthesized in our previous study [25]. This data is presented in Table 1.

After 3 h of stirring, clear reaction mixtures were left for slow solvent evaporation. In the case of Cu(II) propionate—imidazole synthesis, the complex precipitated immediately after adding the imidazole solution. As a result, the molar ratio of Cu(II) and imidazole is in this case different than previously assumed. After 3 h, the precipitate was filtered and washed several times with distilled water. In the next step, the formulas of all synthesized complexes were established and their properties were investigated.

## 3. Results and Discussion

### 3.1. Atomic Absorption Spectrometry

This technique allowed us to determine the contents of metal cations in investigated compounds, which confirms obtaining the compounds that are described by the presented formulas. Table 2 presents measured values in comparison with theoretical ones.

### 3.2. FTIR Spectra

The FTIR spectra of synthesized complexes prove their purity and allow establishing the manner in which carboxylate anions and imidazole bind the metal cation. All analyses have been performed in a manner analogous to the previously studied compounds [25].

**Figure 3:**
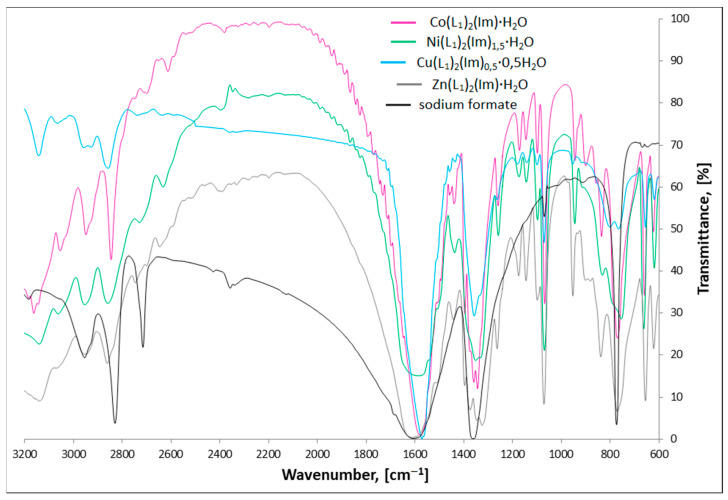
FTIR spectra of studied M(II) formate-imidazole complexes and sodium formate.

**Figure 4:**
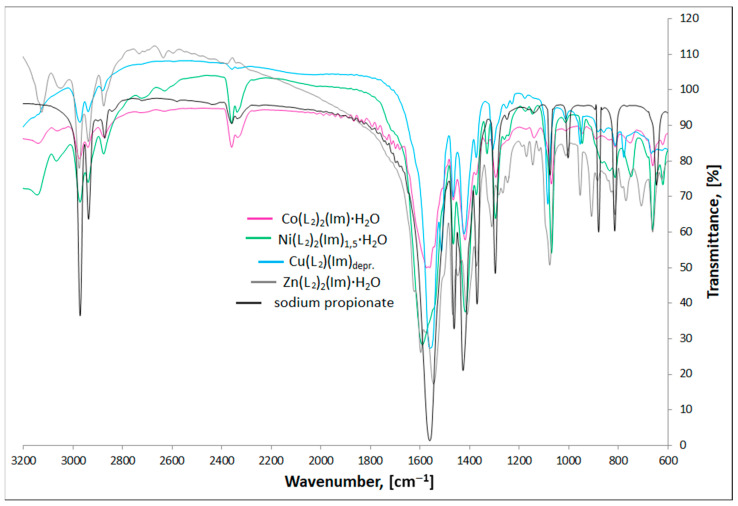
FTIR spectra of studied M(II) propionate-imidazole complexes and sodium propionate.

Figure 3 and Figure 4 present the FTIR spectra of M(II) formate-imidazole and M(II) propionate-imidazole complexes, respectively. The FTIR spectra of sodium formate and sodium propionate have also been added in order to determine how formate and propionate anions coordinate metal centres, according to the spectroscopic criteria described by Nakamoto [29] and Alcock and co-authors [30].

The most important modes observed for studied complexes, sodium formate, and sodium propionate are presented in Table 3.

Stretching *ν*(NH) bands can be found in the range 3139–3037 cm^−1^. It is important to notice that these bands are not observed in the Cu(L_2_)(Im)_d_ spectrum, which is a proof of deprotonation of the NH group in the imidazole molecule for this compound. Stretching (CH)_alifat._ bands can be found in their characteristic area, 2973–2826 cm^−1^. In the region, 955–746 cm^−1^, we can observe a characteristic band that can be ascribed to π(CH) and δ(imidazole ring) vibrations. Recognizing the *ν*(COO)_as._ and *ν*(COO)_sym._ bands allowed us to calculate Δ*ν*(COO) values, according to the formula: Δ*ν*(COO) = *ν*(COO)_as._−*ν*(COO)_sym._ Based on spectroscopic criteria. Nakamoto [29], Alcock and co-authors [30] compared values of separation of *ν*(COO)_as._ And *ν*(COO)_sym._ frequencies of studied compounds with analogous bands found in the spectra of appropriate carboxylate sodium salts. The Δ*ν*(COO) values characterize the nature of metal-carboxylate bond. When Δ*ν*_Na_ > Δ*ν*_complex_, the carboxylate group is considered to be a bidentate chelating ligand, in case of Δ*ν*_Na_ < Δ*ν*_complex_, it coordinates as monodentate ligand and for Δ*ν*_Na_ ≈ Δ*ν*_complex_, it acts as a bidentate-bridging donor [29,30]. On this basis, we can say that in the case of Co(L_1_)_2_(Im)⸱H_2_O, Ni(L_1_)_2_(Im)_1.5_⸱H_2_O, Cu(L_1_)_2_(Im)_0.5_⸱0.5H_2_O, Co(L_2_)_2_(Im)⸱H_2_O, Cu(L_2_)(Im)_d_, and Zn(L_2_)_2_(Im)⸱H_2_O compounds, the carboxylate groups bind the metal centres in a bidentate-bridging manner. In the case of Zn(L_1_)_2_(Im)⸱H_2_O, we can find both higher and lower band separation values in comparison with sodium salt, which indicates two ways of binding the zinc cation: monodentate and bidentate-chelating. Analyzed values suggest the monodentate way of binding in the case of the Ni(L_2_)_2_(Im)_1.5_⸱H_2_O compound.

### 3.3. Thermogravimetric Studies in Air

#### 3.3.1. Thermolysis of M(II) Formate Complexes with Imidazole

Thermal properties have been studied using the TG-DTG method under air atmosphere. All eight compounds are stable at room temperature and decompose gradually when heated. 

**Figure 5:**
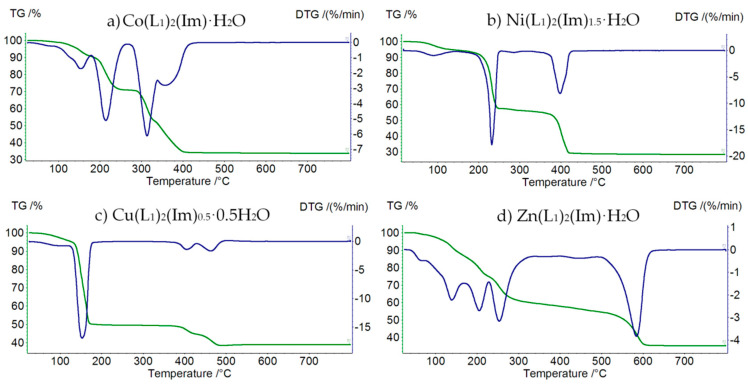
TG (green) and DTG (blue) curves of decomposition of: (**a**) Co(L_1_)_2_(Im)⸱H_2_O, (**b**) Ni(L_1_)_2_(Im)_1.5_⸱H_2_O, (**c**) Cu(L_1_)_2_(Im)_0.5_⸱0.5 H_2_O, and (**d**) Zn(L_1_)_2_(Im)⸱H_2_O.

Thermolysis of Co(L_1_)_2_(Im)⸱H_2_O begins at 50 °C (Figure 5a, Table 4). First step of decomposition is dehydration (mass loss: found. 8.0%; calc. 7.67%). In the temperature range 170–280 °C we observe partial thermolysis of imidazole molecule (mass loss: found. 21.5%; calc. 21.72%). Horizontal mass appears at 450 °C with CoO as a solid decomposition product (mass loss: found. 38.0%; calc. 38.74%).

Ni(L_1_)_2_(Im)_1.5_⸱H_2_O is thermally stable up to 50 °C (Figure 5b, Table 5). In the first step a water molecule is released (mass loss: found. 6.0%; calc. 6.70%). Above 170 °C, decomposition of imidazole begins (mass loss: found. 38.0%; calc. 37.99%). In the temperature range 310–600 °C, the thermodestruction of formate ions takes place (mass loss: found. 27.0%; calc. 27.54%). The final product of decomposition is NiO.

Cu(L_1_)_2_(Im)_0.5_⸱0.5H_2_O starts to decompose at 40 °C (Figure 5c, Table 6). Additionally, in this case, the first step of decomposition is dehydration (mass loss: found. 5.0%; calc. 4.58%). In the temperature range 140–280 °C, we observe the thermolysis of organic molecule, as well as partial decomposition of formate ions (mass loss: found. 46.0%; calc. 45.93%). In the temperature range 280–560 °C, the total decomposition of formates takes place (mass loss: found. 9.5%; calc. 9.03%). Above 560 °C, the horizontal mass for CuO appears.

First step of decomposition of the Zn(L_1_)_2_(Im)⸱H_2_O compound (Figure 5d, Table 7) is dehydration connected with the decomposition of the imidazole molecule in the temperature range 50–270 °C (mass loss: found. 6.0%; calc. 6.70%). In the temperature range 270–620 °C, we observe the thermodesctruction of formate ions (mass loss: found. 30.0%; calc. 30.65%). The final product of decomposition is ZnO.

#### 3.3.2. Thermolysis of M(II) Propionate Complexes with Imidazole

**Figure 6:**
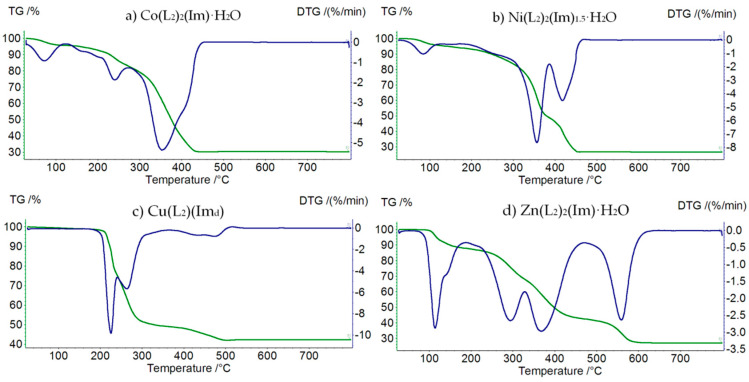
TG (green) and DTG (blue) curves of decomposition of: (**a**) Co(L_2_)_2_(Im)⸱H_2_O, (**b**) Ni(L_2_)_2_(Im)_1.5_⸱H_2_O, (**c**) Cu(L_2_)(Im_d_), and (**d**) Zn(L_2_)_2_(Im)⸱H_2_O.

Decomposition of Co(L_2_)_2_(Im)⸱H_2_O starts at 40 °C (Figure 6a, Table 8). At this temperature, a water molecule is released (mass loss: found. 5.0%; calc. 6.19%). In the temperature range 140–330 °C, the thermodestruction of the imidazole molecule takes place (mass loss: found. 23.0%; calc. 23.38%). In the next step, up to 440 °C, we observe the destruction of propionate anions (mass loss: found. 42.0%; calc. 41.95%). Above 440 °C, horizontal mass for Co_2_O_3_ appears.

Ni(L_2_)_2_(Im)_1.5_⸱H_2_O is thermally stable up to 60 °C (Figure 6b, Table 9). Additionally, in this case, the first step of decomposition is dehydration (mass loss: found. 5.0%; calc. 5.54%). In the next step, in the temperature range 140–370 °C, we can observe the thermolysis of imidazole and partial thermolysis of propionate ions (mass loss: found. 42.5%; calc. 42.67%). In this case, the final product of decomposition is Ni_2_O_3_, formed in the final step (mass loss: found. 26.5%; calc. 26.34%).

Decomposition of Cu(L_2_)(Im_d_) begins at 160 °C (Figure 6c, Table 10). In the temperature range 160–340 °C, the total thermodestruction of imidazole and partial thermodestruction of propionate anions occurs (mass loss: found. 49.5%; calc. 50.86%). Above 340 °C, we observe the total decomposition of propionate anions (mass loss: found. 9.0%; calc. 10.27%). This process stops at 540 °C, when the final solid product of decomposition is formed—CuO.

In the case of Zn(L_2_)_2_(Im)⸱H_2_O (Figure 6d, Table 11), the first step of decomposition is dehydration, occurring at a temperature range of 80–130 °C (mass loss: found. 7.0%; calc. 6.05%). In the second step, up to 320 °C, the imidazole molecule is released (mass loss: found. 23.0%; calc. 22.88%). The last step takes place in the temperature range 320–610 °C and is associated with the themodestruction of propionate anions (mass loss: found. 43.5%; calc. 43.73%). The final solid product of the thermolysis of this compound is ZnO.

### 3.4. Catalytic Activity

**Figure 7:**
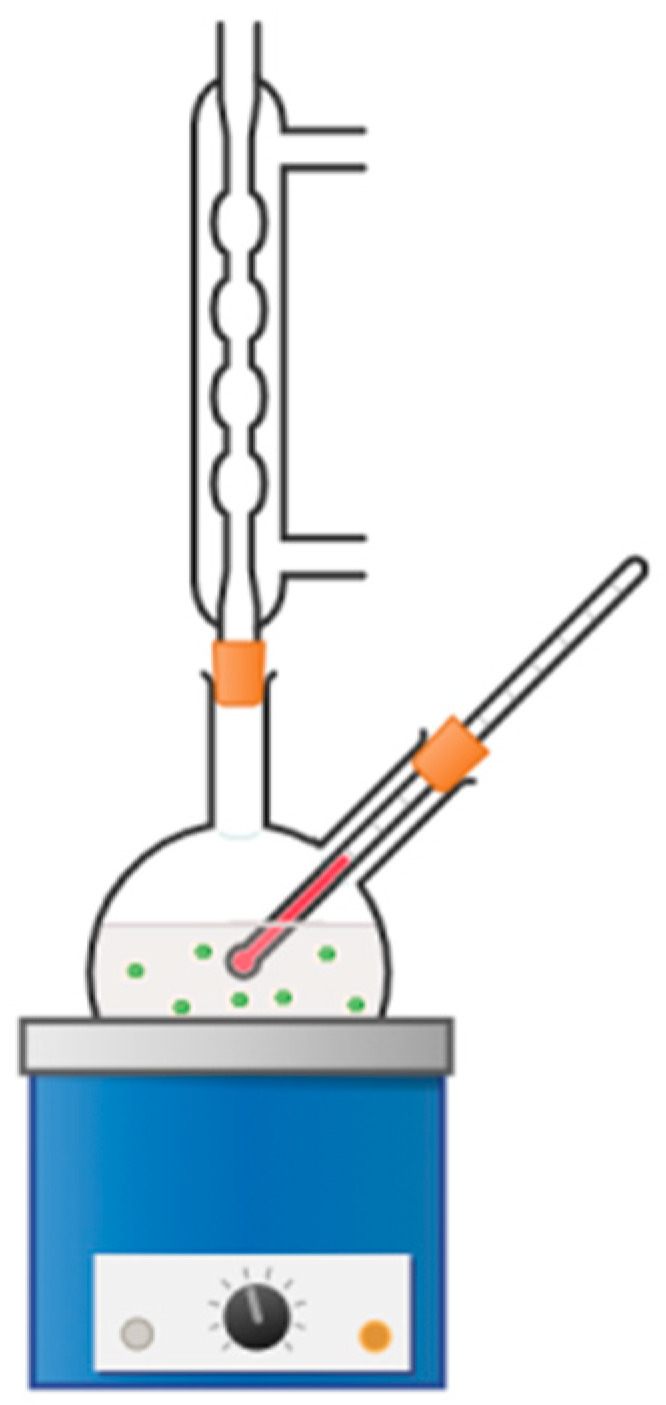
Scheme of the apparatus used for carrying out the reaction of styrene oxidation. The molar ratio of reagents was: C_2_H_3_N:H_2_O_2_:C_8_H_8_ = 1:1:1. After reaching 60 °C, 10 mg of the catalyst was added. Reaction was then carried out for 2 h. After that time, samples for gas chromatography were probed.

Figure 7 presents the scheme of the apparatus used for carrying out the reaction of styrene oxidation described in the Materials and Methods section.

**Figure 8:**
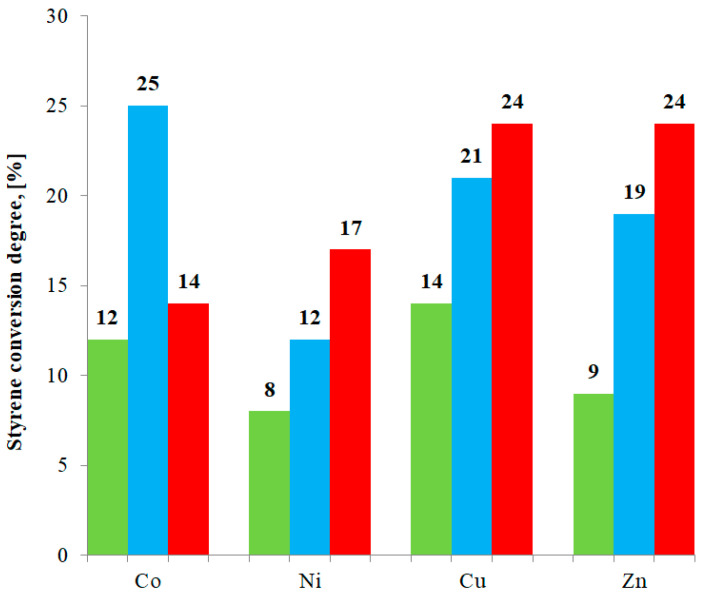
Styrene conversion degree for investigated compounds. Green bars: M(II) formate-imidazole complexes; blue bars: Previously studied M(II) acetate-imidazole complexes [25]; and red bars: M(II) propionate-imidazole complexes.

Table 12 and Figure 8 present conversion degrees for eight described compounds (M(II) formate or propionate complexes with imidazole) as well as for four previously studied compounds [25] (M(II) acetate complexes with imidazole). In case of studied compounds, the main product of oxidation is carbon dioxide.

## 4. Conclusions

In this work, we presented a synthesis procedure, as well as an investigation of properties of eight new compounds: Co(L_1_)_2_(Im)⸱H_2_O, Ni(L_1_)_2_(Im)_1.5_⸱H_2_O, Cu(L_1_)_2_(Im)_0.5_⸱0.5H_2_O, Zn(L_1_)_2_(Im)⸱H_2_O, Co(L_2_)_2_(Im)⸱H_2_O, Ni(L_2_)_2_(Im)_1.5_⸱H_2_O, and Cu(L_2_)(Im)_d_ determine the way organic ligands bind metal centers. It also allowed us to confirm the deprotonation of an imidazole molecule in the case of a Cu(L_2_)(Im)_d_ compound. All eight compounds form solids that are stable at room temperature and decompose gradually when heated. Taking into consideration the thermal stability of investigated compounds, we can say that the carbon chain length had very little effect on this aspect. In all cases, the solid products of decomposition are metal oxides. Of the eight synthesized compounds, Cu(L_2_)(Im_d_) and Zn(L_2_)_2_(Im)⸱H_2_O exhibited the highest catalytic activity. In all cases, M(II) formate-imidazole complexes showed the lowest activity. This study also allowed us to draw some valuable conclusions concerning the relationship between the carbon chain length and catalytic activity. For all Ni(II), Cu(II), and Zn(II) compounds, an increase of the carbon chain length went hand in hand with the increase of conversion degree. In case of Co(II) compounds, Co(Ac)_2_(Im)⸱H_2_O showed the highest activity. The remaining Co(II) compounds exhibited lower activity.

## Figures and Tables

**Table 1:** Formulas of the synthesized M(II) formate and propionate complexes with imidazole and previously synthesized M(II) acetate complexes with imidazole [25].

Co(L_1_)_2_(Im)⸱H_2_O	Co(Ac)_2_(Im)⸱H_2_O	Co(L_2_)_2_(Im)⸱H_2_O
Ni(L_1_)_2_(Im)_1.5_⸱H_2_O	Ni(Ac)_2_(Im)_1,5_⸱2H_2_O	Ni(L_2_)_2_(Im)_1,5_⸱H_2_O
Cu(L_1_)_2_(Im)_0.5_⸱0.5H_2_O	Cu_2_(Ac)_4_(Im)	Cu(L_2_)(Im_d_)
Zn(L_1_)_2_(Im)⸱H_2_O	Zn(Ac)_2_(Im)⸱H_2_O	Zn(L_2_)_2_(Im)⸱H_2_O

**Table 2:** Measured and theoretical percentage M(II) contents for investigated compounds.

Compound	M(II) Content [%]	
	Measured	Theoretical
Co(L_1_)_2_(Im)⸱H_2_O	25.26	25.07
Ni(L_1_)_2_(Im)_1.5_⸱H_2_O	21.66	21.83
Cu(L_1_)_2_(Im)_0.5_⸱0.5H_2_O	32.60	32.32
Zn(L_1_)_2_(Im)⸱H_2_O	27.19	27.07
Co(L_2_)_2_(Im)⸱H_2_O	20.09	20.24
Ni(L_2_)_2_(Im)_1.5_⸱H_2_O	19.01	18.06
Cu(L_2_)(Im)_d_	32.14	31.05
Zn(L_2_)_2_(Im)⸱H_2_O	22.07	21.97

**Table 3:** FTIR modes (cm^−1^) observed for studied M(II) formate- or propionate-imidazole complexes, and for sodium formate and sodium propionate.

	*ν*(NH)	*ν*(CH)_aliphat._	π(CH), δ(Imidazole Ring)	*ν*(COO)_as._	*ν*(COO)_sym._	Δ*ν*(COO)
Sodium formate	-	2946, 2826	-	1585	1350	235
Co(L_1_)_2_(Im)⸱H_2_O	3158, 3139	2942, 2843	943, 884, 834, 767	1570	1339, 1324	231, 246
Ni(L_1_)_2_(Im)_1.5_⸱H_2_O	3129	2939, 2845	943, 825, 765	1571	1331	240
Cu(L_1_)_2_(Im)_0.5_⸱0.5H_2_O	3135	2939, 2917, 2849	951, 794, 759	1572	1351	221
Zn(L_1_)_2_(Im)⸱H_2_O	3126	2941, 2854	955, 887, 935, 768	1605	1386, 1355, 1328, 1317	219, 250, 277, 288
Sodium propoionate	-	2973, 2939, 2869	-	1565	1461, 1424	89, 141
Co(L_2_)_2_(Im)⸱H_2_O	3127	2970, 2934, 2868	943, 899, 822, 811, 746	1566	1476, 1415	90, 151
Ni(L_2_)_2_(Im)_1.5_⸱H_2_O	3136, 3055	2969, 2936, 2871	944, 852, 819, 812, 746	1584	1464, 1414	120, 170
Cu(L_2_)(Im)_d_	-	2969, 2934, 2874	953, 879, 855, 811, 777	1550	1476, 1418	74, 132
Zn(L_2_)_2_(Im)⸱H_2_O	3122, 3037	2973, 2941, 2873	953, 906, 880, 823, 812, 777, 767	1542	1467, 1408	75, 134

**Table 4:** TG-DTG analysis data of decomposition of Co(L_1_)_2_(Im)⸱H_2_O.

Compound/Solid Intermediate	Temperature Range, [°C]	Mass Loss, [%]		Solid Intermediate/Final Residue
		Found	Calculated	
Co(L_1_)_2_(Im)⸱H_2_O	50–170	8.0	7.67	Co(L_1_)_2_(Im)
Co(L_1_)_2_(Im)	170–280	21.5	21.72	Co(L_1_)_2_(Im)_0.25_
Co(L_1_)_2_(Im)_0.25_	280–700	38.0	38.74	CoO

**Table 5:** TG-DTG analysis data of decomposition of Ni(L_1_)_2_(Im)_1.5_⸱H_2_O.

Compound/Solid Intermediate	Temperature Range, [°C]	Mass Loss, [%]		Solid Intermediate/Final Residue
		Found	Calculated	
Ni(L_1_)_2_(Im)_1.5_⸱H_2_O	50–170	6.0	6.70	Ni(L_1_)_2_(Im)_1.5_
Ni(L_1_)_2_(Im)_1.5_	170–310	38.0	37.99	Ni(L_1_)_2_
Ni(L_1_)_2_	310–600	27.0	27.54	NiO

**Table 6:** TG-DTG analysis data of decomposition of Cu(L_1_)_2_(Im)_0.5_⸱0.5H_2_O.

Compound/Solid Intermediate	Temperature Range, [°C]	Mass Loss. [%]		Solid Intermediate/Final Residue
		Found	Calculated	
Cu(L_1_)_2_(Im)_0.5_⸱0.5H_2_O	40–140	5.0	4.58	Cu(L_1_)_2_(Im)_0.5_
Cu(L_1_)_2_(Im)_0.5_	140–280	46.0	45.93	Cu(L_1_)_0.75_
Cu(L_1_)_0.75_	280–560	9.5	9.03	CuO

**Table 7:** TG-DTG analysis data of decomposition of Zn(L_1_)_2_(Im)⸱H_2_O.

Compound/Solid Intermediate	Temperature Range, [°C]	Mass Loss. [%]		Solid Intermediate/Final Residue
		Found	Calculated	
Zn(L_1_)_2_(Im)⸱H_2_O	50–270	6.0	6.70	Zn(L_1_)_2_
Zn(L_1_)_2_	270–620	30.0	30.65	ZnO

**Table 8:** TG-DTG analysis data of decomposition of Co(L_2_)_2_(Im)⸱H_2_O.

Compound/Solid Intermediate	Temperature Range, [°C]	Mass Loss. [%]		Solid Intermediate/Final Residue
		Found	Calculated	
Co(L_2_)_2_(Im)⸱H_2_O	40–120	5.0	6.19	Co(L_2_)_2_(Im)
Co(L_2_)_2_(Im)	140–330	23.0	23.38	Co(L_2_)_2_
Co(L_2_)_2_	330–440	42.0	41.95	Co_2_O_3_

**Table 9:** TG-DTG analysis data of decomposition of Ni(L_2_)_2_(Im)_1.5_⸱H_2_O.

Compound/Solid Intermediate	Temperature Range, [°C]	Mass Loss. [%]		Solid Intermediate/Final Residue
		Found	Calculated	
Ni(L_2_)_2_(Im)_1.5_⸱H_2_O	60–140	5.0	5.54	Ni(L_2_)_2_(Im)_1.5_
Ni(L_2_)_2_(Im)_1.5_	140–370	42.5	42.67	Ni(L_2_)_1.5_
Ni(L_2_)_1.5_	370–560	26.5	26.34	Ni_2_O_3_

**Table 10:** TG-DTG analysis data of decomposition of Cu(L_2_)(Im_d_).

Compound/Solid Intermediate	Temperature Range, [°C]	Mass Loss. [%]		Solid Intermediate/Final Residue
		Found	Calculated	
Cu(L_2_)(Im_d_)	160–340	49.5	50.86	Cu(L_2_)_0.5_
Cu(L_2_)_0.5_	340–540	9.0	10.27	CuO

**Table 11:** TG-DTG analysis data of decomposition of Zn(L_2_)_2_(Im)⸱H_2_O.

Compound/Solid Intermediate	Temperature Range, [°C]	Mass Loss. [%]		Solid Intermediate/Final Residue
		Found	Calculated	
Zn(L_2_)_2_(Im)⸱H_2_O	80–130	7.0	6.05	Zn(L_2_)_2_(Im)
Zn(L_2_)_2_(Im)	130–320	23.0	22.88	Zn(L_2_)_2_
Zn(L_2_)_2_	320–610	43.5	43.73	ZnO

**Table 12:** Conversion degrees for eight described compounds (M(II) formate or propionate complexes with imidazole) and for four previously studied compounds [25] (M(II) acetate complexes with imidazole).

Compound	Styrene Conversion Degree [%]
Co(L_1_)_2_(Im)⸱H_2_O	12
Ni(L_1_)_2_(Im)_1.5_⸱H_2_O	8
Cu(L_1_)_2_(Im)_0.5_⸱0.5H_2_O	14
Zn(L_1_)_2_(Im)⸱H_2_O	9
Co(Ac)_2_(Im)⸱H_2_O	25
Ni(Ac)_2_(Im)_1.5_⸱2H_2_O	12
Cu_2_(Ac)_4_(Im)	21
Zn(Ac)_2_(Im)⸱H_2_O	19
Co(L_2_)_2_(Im)⸱H_2_O	14
Ni(L_2_)_2_(Im)_1.5_⸱H_2_O	17
Cu(L_2_)(Im_d_)	24
Zn(L_2_)_2_(Im)⸱H_2_O	24

## Data Availability

Not applicable.

## References

[1] Shalini K., Sharma P.K., Kumar N. (2010). Imidazole and its biological activities: A review. Der. Chem. Sin..

[2] Siwach A., Verma P.K. (2021). Synthesis and therapeutic potential of imidazole containing compounds. BMC Chem..

[3] Gupta V., Kant V. (2013). A Review on Biological Activity of Imidazole and Thiazole Moieties and their Derivatives. Sci. Int..

[4] De Luca L. (2006). Naturally occurring and synthetic imidazoles: Their chemistry and their biological activities. Curr. Med. Chem..

[5] Sharma D., Narasimhan B., Kumar P., Judge V., Narang R., De Clercq E., Balzarini J. (2009). Synthesis, antimicrobial and antiviral evaluation of substituted imidazole derivatives. Eur. J. Med. Chem..

[6] Sharma P., LaRosa C., Antwi J., Govindarajan R., Werbovetz K.A. (2021). Imidazoles as Potential Anticancer Agents: An Update on Recent Studies. Molecules..

[7] Mlostoń G., Celeda M., Poper W., Kowalczyk M., Gach-Janczak K., Janecka A., Jasiński M. (2020). Synthesis, Selected Transformations, and Biological Activity of Alkoxy Analogues of Lepidilines A and C. Materials.

[8] Gholami Z., Tišler Z., Rubáš V. (2021). Recent advances in Fischer-Tropsch synthesis using cobalt-based catalysts: A review on supports, promoters, and reactors. Catal. Rev..

[9] Mirzaei A.A., Arsalanfar M., Bozorgzadeh H.R., Samimi A. (2014). A review of Fischer-Tropsch synthesis on the cobalt based catalysts. Phys. Chem. Res..

[10] Zhou H., Song J., Fan H., Zhang B., Yang Y., Hu J., Zhu Q., Han B. (2014). Cobalt catalysts: Very efficient for hydrogenation of biomass-derived ethyl levulinate to gammavalerolactone under mild conditions. Green Chem..

[11] Zybert M., Tarka A., Mierzwa B., Raróg-Pilecka W. (2017). Cobalt-lanthanum catalyst precursors for ammonia synthesis: Determination of calcination temperature and storage conditions. Pol. J. Chem. Tech..

[12] Izumi Y., Imaida M., Fukawa H., Akabori S. (1963). Asymmetric Hydrogenation with Modified Raney Nickel. Studies on Modified Hydrogenation Catalyst. Bull. Chem. Soc. Jpn..

[13] Harada T., Izumi Y. (1978). Improved modified Raney Nickel catalyst for enantioface-differentiating (asymmetric) hydrogenation of methyl acetoacetate. Chem. Lett..

[14] Studentschnig A.F.H., Schober S., Mittelbach M. (2013). Conversion of Crude Palm Oil into Hydrocarbons over Commercial Raney Nickel. Energy Fuels.

[15] Abudayyeh A.M., Schott O., Feltham H.L.C., Hanan G.S., Brooker S. (2021). Copper catalysts for photo- and electro-catalytic hydrogen production. Inorg. Chem. Front..

[16] Bluhm H., Hävecker M., Knop-Gericke A., Kleimenov E., Schlogl R. (2004). Methanol Oxidation on a Copper Catalyst Investigated Using in Situ X-ray Photoelectron Spectroscopy. J. Phys. Chem. B.

[17] Górecka S., Pacultová K., Fridrichová D., Górecki K., Bílková T., Žebrák R., Obalová L. (2021). Catalytic Oxidation of Ammonia over Cerium-Modified Copper Aluminium Zinc Mixed Oxides. Materials.

[18] Nisa R.U., Mahmood T., Ludwig R., Ayub K. (2016). Theoretical mechanistic investigation of zinc(II) catalyzed oxidation of alcohols to aldehydes and esters. RSC Adv..

[19] Cheung E., Alberti C., Enthaler S. (2020). Chemical Recycling of End-of-Life Poly(lactide) via Zinc-Catalyzed Depolymerization and Polymerization. ChemistryOpen.

[20] Adam F., Iqbal A. (2010). The oxidation of styrene by chromium–silica heterogeneous catalyst prepared from rice husk. Chem. Eng. J..

[21] Kumar S.B., Mirajkar S.P., Pais G.C.G., Kumar P., Kumar R. (1995). Epoxidation of Styrene over a Titanium Silicate Molecular Sieve TS1 Using Dilute H_2_O_2_ as Oxidizing Agent. J. Catal..

[22] Gao D., Gao Q. (2007). Selective oxidation of styrene to benzaldehyde over VSB-5 and isomorphously substituted cobalt VSB-5. Catal. Commun..

[23] Aberkouks A., Mekkaoui A.A., Boualy B., El Houssame S., Ali M.A., El Firdoussi L. (2018). Selective oxidation of styrene to benzaldehyde by Co-Ag codoped ZnO catalyst and H_2_O_2_ as oxidant. Adv. Mater. Sci. Eng..

[24] Titinchi S.J., VonWillingh G., Abbo H.S., Prasad R. (2015). Tri- and tetradentate copper complexes: A comparative study on homogeneous and heterogeneous catalysis over oxidation reactions. Catal. Sci. Technol..

[25] Czylkowska A., Rogalewicz B., Raducka A., Błaszczyk N., Maniecki T., Wieczorek K., Mierczyński P. (2020). Synthesis, Spectroscopic, Thermal and Catalytic Properties of Four New Metal (II) Complexes with Selected N- and O-Donor Ligands. Materials.

[26] Gueorguiev G.K., Pacheco J.M. (2003). Shapes of cagelike metal carbide clusters: First-principles calculations. Phys. Rev. B.

[27] Dos Santos R.B., Rivelino R., de Brito Mota F., Gueorguiev G.K., Kakanakova-Georgieva A. (2015). Dopant species with Al–Si and N–Si bonding in the MOCVD of AlN implementing trimethylaluminum, ammonia and silane. J. Phys. D Appl. Phys..

[28] Tavares S.R., Vaiss V.S., Novais Antunes F.P., Fonseca C.G., Nangoi I.M., Moraes P.I.R., Soares C.V., Haddad J.F.S., Lima L.L., Silva B.N.N. (2018). DFT calculations for structural prediction and applications of intercalated lamellar compounds. Dalton Trans..

[29] Nakamoto K. (2009). Infrared and Raman Spectra of Inorganic and Coordination Compounds.

[30] Alcock N.W., Tracy V.M., Waddington T.C. (1976). Acetates and acetato-complexes. Part 2. Spectroscopic studies. J. Chem. Soc. Dalton Trans..

